# Vaccination to prevent human papillomavirus infections: From promise to practice

**DOI:** 10.1371/journal.pmed.1002325

**Published:** 2017-06-27

**Authors:** Paul Bloem, Ikechukwu Ogbuanu

**Affiliations:** World Health Organization, Geneva, Switzerland

## Abstract

In an essay, Paul Bloem and Ikechukwu Ogbuanu discuss the public health implications of HPV vaccination.

**Summary points**

- A large proportion of the disease burden related to infection with human papillomavirus (HPV), the most common sexually transmitted infection, can be prevented through vaccination.
- There is inequity in access to HPV vaccines, and populations with the largest burden of HPV-related diseases have the least access to the vaccines.
- Affordability and sustainable financing of HPV vaccination are barriers to introduction in low- and middle-income countries because of the relatively high cost of the vaccine and its delivery to a nontraditional target population.

## Global burden of HPV-related diseases

Human papillomavirus (HPV) is the most common sexually transmitted infection (STI) and can cause HPV-related cancers, most of which are cervical cancers, and genital warts. Although the majority of HPV infections are asymptomatic with spontaneous resolution, disease may result from persistent infection with high-risk HPV genotypes. While infection with a high-risk oncogenic HPV type is the underlying cause of virtually all cases of cervical cancer, most infections with high-risk HPV types do not lead to cancer. This is because infection persists only in a small percentage of people; only a small proportion of these persistent infections progress to precancer, and, of these, even fewer lead to invasive cancer [1].

Two HPV types (16 and 18) cause 70% of cervical cancers and contribute to other, less prevalent, noncervical cancers. HPV infections are responsible for nearly all of the 528,000 estimated cervical cancer cases in women each year, accounting for nearly 266,000 deaths yearly [2]. High age-standardised rates of cervical cancer are observed in parts of sub-Saharan Africa, Latin America, and Southeast Asian regions. Low rates are seen in the Eastern Mediterranean region as well as in North America, Western Europe, and Australia [2]. HIV-positive women have a 5-fold elevated risk of developing cervical cancer.

Two additional HPV types (6 and 11) are responsible for up to 90% of anogenital warts [1]. Anogenital warts are a common manifestation of HPV infection among young men and women. The overall reported annual incidence (for males and females combined) of anogenital warts (including new and recurrent) ranges from 160 to 289 per 100,000, with a median of 194.5 per 100,000. The estimated median annual incidence of new anogenital warts is 137 per 100,000 among males and 120.5 per 100,000 among females [3]. Rates are higher in HIV-infected populations such as those in sub-Saharan Africa (2.4% to 14%) [4].

HPV infections are also associated with other cancers, including vaginal, vulvar, penile, anal, and oropharyngeal cancers. Of the estimated 27,000 cases of anal cancers globally, 88% (24,000) were caused by HPV infections, predominantly by HPV types 16 and 18 [5].

## HPV vaccination promises large impact

For the first time in the history of STI prevention and control programmes, an intervention is available to prevent one of the most common STIs, HPV infection. Vaccines to prevent infection by high-risk oncogenic HPV types (16 and 18) and HPV types that cause anogenital warts (6 and 11) have been in use since 2006. HPV vaccines are most effective when administered before the onset of sexual activity. By preventing HPV infection, they could greatly reduce the morbidity and mortality from cervical cancers [1]. Early-impact studies from high-income settings with high vaccination coverage have shown a reduction of up to 30% in cervical intraepithelial neoplasia grade 2+ (CIN2+) in 15 to 19 year old girls [6]. Where the quadrivalent vaccine was used, data are showing a remarkable impact on the incidence of genital warts, including among unvaccinated males through herd protection [7].

There are currently 3 HPV vaccines available. The bi- and quadrivalent vaccines have been on the market since 2006; both vaccines protect against the most common oncogenic genotypes of HPV (types 16 and 18). In 2014, a 9-valent vaccine was licensed, which protects against 5 additional oncogenic HPV types, in addition to types 16 and 18. The quadri- and 9-valent vaccines also protect against HPV types 6 and 11, which cause genital warts; both are licensed for use in males [8]. The available vaccines are cost-effective in most settings for the prevention of cervical cancer [1] and have the potential to significantly reduce the burden of anogenital warts.

Originally requiring 3 doses for full protection, the HPV vaccination schedule was revised in 2014. Adolescents 14 years or younger now require just 2 doses for full protection, with an interval of at least 6 months and up to 12 or 15 months between doses [1]. WHO recommends the HPV vaccine for 9- to 14-year-old girls before the onset of sexual activity. To accelerate its impact during initial introduction into national immunisation programmes, vaccination of a multicohort of eligible girls aged 9 to 14 years (or, if affordable, up to 18 years of age) is also recommended [8].

## Inequity in access to HPV vaccination

Given 10 years of availability and the documented effectiveness of HPV vaccines, there remain wide global disparities in access to this intervention. Globally, by the end of 2016, nearly 70 countries (or 35% of all countries) have introduced the vaccine into their national immunisation schedule, either nationally or in a part of the country [9,10]. Unfortunately, full-dose vaccine coverage remains low in many settings. It has been estimated that between 2006 and the end of 2014, only 1.4% of the global population of women 10 to 20 years of age had received the full course of HPV vaccine [11].

Similarly, the countries with the highest morbidity owing to cervical cancer and anogenital warts are also the ones least likely to have introduced the vaccine. By June 2016, 71% of high-income countries, 35% of upper-middle-income countries, 8% of lower-middle-income countries, and 6% of low-income countries had introduced the HPV vaccine [8]. Whereas girls and women in high-income countries have the highest likelihood to be vaccinated, 90% of the global burden of cervical cancer (and probably a similar share of anogenital warts) occurs in middle- and low-income countries [1].

In a recent analysis by WHO, the proportion of girls living in countries with access to publicly funded HPV vaccination differed markedly by region. Only in the Americas and the European region do the majority of girls currently live in countries with access to free HPV vaccination (86% in the Americas and 62% in the European region). This proportion drops to 10% or less in all other regions [8]. With regard to access by gender, of the 70 countries (or provinces) who reported HPV vaccine introduction by the end of 2016, 8 make the vaccines available to adolescent boys in addition to adolescent girls (Australia, Austria, Barbados, Brazil, Canada, Italy, Switzerland, and the United States) [9].

Current WHO recommendations prioritise vaccine access for girls for the purpose of cervical cancer prevention. This recommendation was based on modelling data showing that, for the prevention of cervical cancer, reaching high coverage in girls is more cost-effective than attaining lower coverage in both boys and girls [1]. However, consideration for gender-neutral immunisation should be a country-level decision based on factors such as disease burden, local sexual behaviour patterns, equity concerns, programmatic implications, cost-effectiveness, and affordability. Because of herd protection effects, heterosexual males also benefit from vaccinating girls [8,6]. Since men who have sex with men (MSM) are likely to benefit less from this herd protection effect and also have an elevated risk of HIV infection, some countries have extended HPV vaccination specifically to MSM [9].

## Growing support for HPV introduction among the global health community

The magnitude of the burden of cervical cancer coupled with the importance of anogenital warts and their impact on quality of (sexual) life, as well as the availability of effective and safe vaccines for prevention, have recently led to considerable policy attention in support of the introduction of HPV vaccines. In the context of the Decade of Vaccines, the Global Vaccine Action Plan promotes the introduction of new vaccines, including HPV vaccine [12]. In 2012, the Gavi Alliance opened a window of support for HPV vaccine introduction, with an original target to reach 30 million girls in the least developed countries by 2020 [13]. In addition, more recently, the UN Global Action Plan for the Prevention and Control of Noncommunicable Diseases identified cervical cancer as a priority preventable cancer and HPV vaccination as a key intervention [14]. In 2016, the interagency task team on cancer set up a Joint UN Global Programme for the Prevention of Cervical Cancer to accelerate implementation of comprehensive cervical cancer prevention programmes, including vaccination in high-burden countries. Also in 2016, the World Health Assembly adopted the Global Health Sector Strategies on STIs, which endorse the effectiveness of HPV vaccination as a cost-effective STI prevention strategy and encourage countries to introduce the vaccine and achieve high coverage [15].

In low-income countries, all introductions to date have been supported by donors (vaccine manufacturers, government aid programmes, or the Gavi Alliance). Many of the first wave of countries implemented a Gavi-supported demonstration programme. The demonstration programmes aimed to assess the feasibility and cost implications of different vaccine delivery strategies for adolescent girls, a group that presents unique challenges and opportunities to most immunisation programmes in low-income countries that traditionally focus on infant vaccination [13]. To date, only a small fraction of these Gavi demonstration programmes have resulted in decisions to proceed with national scale-up. The Gavi Alliance has thus far supported national introductions in 4 countries, but this number is projected to increase to 20 countries by 2020.

## Challenges and opportunities

In addition to vaccine cost, a major barrier to HPV vaccine uptake is the lack of experience delivering a 2-dose vaccine to 9- to 14-year-old girls through routine immunisation programmes. Country experiences have shown that high coverage can be achieved in multiple settings. Several countries in different income categories have reported vaccination coverage levels above 80% (e.g., England and Mexico) or 90% (e.g., Malaysia and Rwanda). On the other hand, there are also examples of countries in which full-dose coverage remains below 50% more than 5 years following introduction (e.g., France and the US) [9].

HPV vaccines have been shown to be highly effective in real-world settings. Impact studies have shown significant reductions in the circulation of oncogenic and other genotypes included in the vaccine and reductions in the incidence of genital warts and high-grade precancerous lesions [6]. In addition, after more than 200 million doses have been administered worldwide and following frequent reviews of their safety profiles, the available vaccines continue to show excellent safety [16]. In spite of this, serious challenges remain to improving uptake and coverage of the vaccines among girls and women around the world, particularly in low- and middle-income countries (LMICs).

A key challenge for LMICs is the sustainable financing of HPV vaccine introduction. This is driven by 2 factors—vaccine price and delivery cost. Gavi-eligible countries are relatively (and temporarily) shielded from the high vaccine cost, as they finance the vaccine at a fraction of the Gavi price of $4.50 per dose [13] (so called “co-financing” ranges from $0.20 per dose gradually increasing to 80% of the GAVI price, depending on Gavi eligibility status). However, many countries approaching transition from Gavi support are concerned about rapidly increasing vaccine costs. Similarly, through the Revolving Fund, PAHO countries can access HPV vaccines at a cost of $8.50 per dose [17], which is a key factor behind the high uptake among the middle-income countries (MICs) in that region. The price available to MICs in other regions is often considerably higher, creating a major barrier to uptake [18]. Recent publications challenge the high cost of these life-saving vaccines, alleging that they are overpriced. A recent estimate suggests that the manufacturing cost of the quadrivalent HPV vaccine may be as low as $0.48 to $0.59 per dose [19].

Similar to the vaccine cost, vaccine delivery costs disproportionately impact LMICs. In many LMICs, no platform exists to deliver vaccines to adolescents. In LMICs with high school enrolment and attendance, the target population of girls could be reached through school vaccination programmes. Unfortunately, in countries in which school health programmes are nonexistent, this delivery strategy comes at a higher cost relative to routine delivery in health facilities. Due to these costs, several low-income countries, including Bhutan, have continued to reassess and modify their vaccine delivery model in the years following introduction [20,21]. This underscores the need for programmes to understand the main cost drivers prior to introduction and to develop locally acceptable, affordable, and sustainable delivery strategies.

Another challenge is that the novel target age group makes the HPV vaccine vulnerable to more scrutiny than traditional infant vaccines. Countries of all income groups have had to prepare for and deal with crisis communication related to safety or other concerns in order to reach high levels of acceptance and sustain this through crises. Although HPV vaccines have been well accepted in many settings (indicated by coverage levels >80%), acceptance in some other countries remains a challenge (including Romania, France, and the US) [9]. While overall health systems issues (including the choice of delivery strategies) may explain the low uptake in these countries, vaccine hesitancy spurred by anti-vaccination sentiments, alleged safety issues, and doubts over the added value of the vaccine may also play a role. When safety concerns are not dealt with adequately and in a timely manner, these can seriously jeopardise acceptance and coverage. A prime example is the safety concern in Japan, where in 2013 a small number of cases of chronic regional pain syndrome (CRPS) were reported, which were subsequently shown to be unrelated to vaccination [16,22]. As a result of the ensuing communication crisis, the health ministry suspended the active recommendation of HPV vaccination, resulting in reduced vaccination coverage ever since [9]. More recently, similar alleged safety issues in Denmark also adversely affected population trust in the vaccine. Proactive and immediate efforts by the national programme in Denmark have aimed to restore trust in the HPV vaccine and have resulted in a gradual recovery in coverage levels since 2016 [9].

Although the majority of LMICs introduce the HPV vaccine for cervical cancer control, many do not have a well-performing cervical cancer screening programme for women. Similarly, many LMICs do not have a sustainable preventive health delivery platform for adolescents. The introduction of HPV vaccination could be an opportunity to address both of these deficiencies. For instance, HPV vaccine introduction provides a rare opportunity to build or strengthen school health and adolescent health platforms. These platforms could offer additional preventive and curative health interventions for adolescents. Such interventions could include the provision of tetanus boosters, meningococcal vaccines, or the new dengue vaccine, as relevant, but also deworming tablets, iron and folic acid supplementation, or health education messages, including on sexual and reproductive health, STIs, and HIV [23].

## Conclusion

In the 10 years since its introduction, HPV vaccination has seen many positive developments. A reduced number of doses and more flexible schedules have reduced costs and facilitated programme implementation. Recent clinical trials are assessing the efficacy and duration of protection offered by 1 dose of HPV vaccine, based on recent promising data on efficacy [24, 25]. The HPV vaccine market is also likely to evolve further. The 9-valent vaccine is expected to expand to markets beyond the US. Additional vaccine manufacturers are expected to introduce new and cheaper HPV vaccines. These positive developments are likely to significantly change the global HPV vaccine market in the coming years.

At the currently projected pace of HPV vaccine introduction and uptake, a significant impact of the HPV vaccine on cancer prevention will not be seen until 15 to 20 years postintroduction. Scenarios for faster uptake and impact have been proposed in literature, including combinations of vaccination and HPV DNA testing [26]. Until some of the major barriers, including the price and availability of these interventions, are addressed, such scenarios may remain beyond the reach of LMICs for some time.

Several factors are combining to accelerate uptake and coverage of HPV vaccines among LMICs. Most notable are the increased policy attention to STI and cervical cancer prevention and control, the ongoing support from the Gavi Alliance to low-income countries, and the continued downward pressure on HPV vaccine prices. The potential impact of increased access to this vaccine on STI and cervical cancer prevention is enormous. We are confident that, over the next decade, the HPV vaccine will fulfil its promise and reach its full public health potential through broader availability.

## Abbreviations

CIN2+: cervical intraepithelial neoplasia grade 2+
CRPS: chronic regional pain syndrome
HPV: human papillomavirus
LMICs: low- and middle-income countries
MIC: middle-income country
MSM: men who have sex with men
STI: sexually transmitted infection
